# Population mixing and childhood leukaemia and non-Hodgkin's lymphoma in census wards in England and Wales, 1966–87

**DOI:** 10.1038/sj.bjc.6600275

**Published:** 2002-05-06

**Authors:** H O Dickinson, D M Hammal, J F Bithell, L Parker

**Affiliations:** North of England Children's Cancer Research Unit, Department of Child Health, University of Newcastle, Royal Victoria Infirmary, Queen Victoria Road, Newcastle upon Tyne NE1 4LP, UK; Department of Statistics, University of Oxford, 1 South Parks Road, Oxford OX1 3TG, UK

**Keywords:** childhood leukaemia, population mixing, epidemiology

## Abstract

We found an increased risk of childhood leukaemia with higher levels of inward migration, particularly from outside the region (rate ratio=1.9, 95%CI: 1.2–2.9, *P*<0.01). This significant effect was observed only in urban areas, although a marked but non-significant effect was seen in affluent, rural areas.

*British Journal of Cancer* (2002) **86**, 1411–1413. DOI: 10.1038/sj/bjc/6600275 www.bjcancer.com

© 2002 Cancer Research UK

## 

In a series of studies of rural areas with unusually high levels of inward migration, Kinlen and colleagues have shown that children living in such areas are at increased risk of leukaemia and non-Hodgkin's lymphoma (NHL) (Kinlen, 2000). A further study of population mixing, based on all 403 county districts in England and Wales, showed that the risk of lymphoblastic leukaemia among children increased with increasing levels of migration (Stiller and Boyle, 1996). However, county districts have a median size of about 200 km^2^ and so are likely to include areas which have substantially different levels of population mixing. Analysis using smaller areas, which should be more homogeneous, is therefore of interest.

The aim of this study was to investigate the association between population mixing as measured in census wards (median size 6 km^2^) and the risk of childhood leukaemia and NHL in England and Wales.

## METHODS

We considered all 10 194 cases of leukaemia and NHL registered in children under the age of 15 years in England and Wales between 1966 and 1987. Although ward boundaries changed in a reorganisation of local government in 1974 (Local government act, 1972), population, geographic and socio-demographic data were available for ‘census tracts’, which can be defined in terms of aggregations of both 1971 and 1981 enumeration districts (OPCS, 1985). These census tracts were aggregated such that they corresponded as closely as possible to 1981 wards; suitably weighted sums of the required variables (population, centroid, Townsend score, obtained from the 1971 and 1981 censuses) were computed and cases were assigned to the relevant areal unit on the basis of their address at registration (Dutton *et al*, 1994). The expected number of cases in each ward was estimated using a Poisson regression model which took into account the population at risk in the age groups 0–4, 5–9 and 10–14 years, the Townsend community deprivation score, and adjusted for the nine health regions. This dataset was originally assembled for a study of leukaemia and NHL in relation to proximity to nuclear installations, and fuller details of its construction have been reported (Bithell *et al*, 1994; Dutton *et al*, 1994). Wards within the same county district which had a very small number of child residents (under 100) were merged to form separate units (this affected 12 wards in the City of London and five wards in the Isles of Scilly), resulting in a total of 8786 areal units for analysis.

The dataset was enhanced by obtaining migration data at ward level. The total numbers of residents and migrants (those who had changed address in the year before the census) were extracted from Special Migration Statistics of the 1981 census. Measures of migration at six levels were calculated for each ward – the proportion of incomers from outside: (i) Great Britain, (ii) the region, (iii) the county, (iv) the county district, (v) the ward; and (vi) the proportion of all migrants in the ward, including those moving within the ward. These measures were cumulative: each level included movers at the preceding level. A measure of the diversity of origins of incomers into each ward was also calculated (Stiller and Boyle, 1996). On the basis of the Townsend deprivation score, wards were categorised as affluent (<10-percentile), medium (>=10-percentile and <90-percentile), or deprived (>=90-percentile). A six-category urban/rural indicator for wards was obtained from the 1981 census, classifying each ward as: wholly urban, predominantly urban, mixed urban, mixed rural, predominantly rural or wholly rural. This categorisation was based on matching enumeration districts to the physical boundaries of urban land using information supplied by Ordnance Survey; no comparable classification was available for the 1971 census (OPCS, 1984).

Poisson regression, stratified by Townsend deprivation category, was used to derive rate ratios and their 95% confidence intervals (based on profile likelihoods) in relation to population mixing, which was standardised to have a range from zero to one and treated as a continuous variable, so that rate ratios correspond to a trend in risk from the lowest to the highest levels. Differences in risk in urban and rural areas were investigated and, finally, differences in the effect of population mixing in urban and rural areas were assessed by adding an interaction term. The goodness-of-fit of the resulting statistical models was assessed by Monte Carlo simulation (Bithell *et al*, 1995).

Sensitivity analysis was performed to assess the effect of variation over time in migration data. Firstly, a weighted average of 1981 and 1991 migration data (extracted from Small Area Statistics for 1991) was used. Secondly, as migration data for the 1971 census were available only for a 10% sample of the population, were not reported by the distance over which people had moved and were not available for areas corresponding to 1981 wards, they could not be used. However, as migration in England and Wales, as measured by the census, was about 25% higher in 1971 than in 1981 (Stillwell *et al*, 1992), we carried out a simulation, assuming the mean level of migration in wards in 1971 was 1.25 times the 1981 value but adding a random perturbation (of standard deviation 0.05) to the 1981 data and then using a weighted average of 1971 and 1981 data.

The percentage of cases attributable to population mixing was estimated; this was the difference between the observed number of cases and the number of cases predicted by the statistical model in the absence of population mixing, expressed as a percentage of the observed number of cases (Greenland and Drescher, 1993).

## RESULTS

The number of cases of leukaemia and non-Hodgkin's lymphoma varied from zero in 3896 wards to 11 in four wards. The median rate was eight cases/100 000 person-years and the highest rate was 119 cases/100 000 person-years.

Most migrants moved within a county district (see Table 1

**Table 1:**
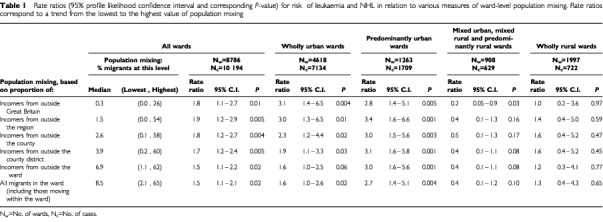
Rate ratios (95% profile likelihood confidence interval and corresponding *P*-value) for risk of leukaemia and NHL in relation to various measures of ward-level population mixing. Rate ratios correspond to a trend from the lowest to the highest value of population mixing

). The pattern of migration was very skewed, with a few wards having proportions of migrants over seven times the median. For movers over greater distances, the pattern became progressively more skewed.

The rate ratios in relation to population mixing are shown in Table 1. Population mixing was associated with an increased risk of leukaemia and NHL, the effect being determined largely by incomers from outside the region (RR=1.9, 95%CI: 1.2–2.9). Inclusion of movers from closer areas reduced the effect, suggesting that these migrants had less effect on the risk of leukaemia and NHL. This was confirmed by consideration of movement only within wards, for which the effect was negligible (RR=1.1, 95%CI: 0.9–1.4, *P*=0.54). However, the increased risk with greater population mixing was consistently observed only in wholly or predominantly urban areas. In mixed urban, mixed rural and predominantly rural areas, which included only 6% of the population, the risk of leukaemia and NHL decreased as population mixing increased; these wards are therefore grouped in Table 1. There was no general association of risk with population mixing in wholly rural areas, although a very marked but non-significant risk was evident in affluent rural wards (for population mixing based on all movers, RR=14.0, 95%CI: 0.3–266, *P*=0.16); these wards included only 0.7% of the total childhood population. A comparison of urban areas (wholly and predominantly urban wards) and rural areas (mixed urban, mixed rural, predominantly and wholly rural wards) showed that, although there was no significant difference between the risk of leukaemia and NHL in urban and rural areas, the effect of population mixing was significantly (*P*<0.02) greater in urban areas than elsewhere. These effects were similar in all regions and changed little when population mixing at each level was weighted by a measure of the diversity of origins of incomers (Stiller and Boyle, 1996). Sensitivity analysis showed that the results were virtually unchanged by use of a weighted average of 1981 and 1991 migration data; when a weighted average of simulated 1971 and known 1981 data was used, the overall pattern of results was similar. Population mixing (based on all movers) accounted for less than 5% and 8% of cases in all wards and urban wards respectively. All models were a satisfactory fit (*P*>0.10).

## DISCUSSION

As the analysis used an existing dataset aggregated over diagnostic groups, age groups and time periods, it was not possible to investigate separately the risk for lymphatic leukaemia, especially that in younger children, which may be more closely related than other childhood leukaemias to population mixing (Dickinson and Parker, 1999), or to allow for changes over time in population mixing or urbanisation. However, sensitivity analysis suggested that our findings were robust to temporal changes in migration. Urbanisation would have resulted in misclassification of the urban/rural indicator largely in wards classified as ‘mixed urban, mixed rural and predominantly rural’, where we found no significant effect of population mixing.

Despite these limitations, our findings of a significantly increasing risk of leukaemia and NHL with increasing community population mixing in ward of residence at diagnosis, which was more marked for movers from a greater distance, confirm those of Stiller and Boyle (1996). However, we found that this pattern of risk was restricted to urban areas and possibly affluent, rural areas.

Dickinson and Parker found a much more marked effect of community population mixing around the time of birth on the risk of these diseases in Cumbrian-born children (RR= 6.8, 95%CI: 1.9–24) than that found in the present study, suggesting that exposure in early life may be more directly related to risk than exposure nearer to the time of diagnosis (Dickinson and Parker, 1999). They also found that migration of a child's parents was significantly associated with the child's susceptibility to leukaemia and NHL.

Further studies are required to elucidate the complex relationship between individual susceptibility, community population mixing and other characteristics of the environment, including differences between urban and rural areas.
